# The Changing Landscape of Pediatric Cardiology Fellowship Applications: An Update on Objective Metrics and Current Evaluation Practices

**DOI:** 10.7759/cureus.103065

**Published:** 2026-02-05

**Authors:** William B Kyle, Tam T Doan, James C Wilkinson

**Affiliations:** 1 Pediatric Cardiology, Baylor College of Medicine, Houston, USA

**Keywords:** eras, fellowship evaluation form, letters of recommendation, medical school class rank, pediatric cardiology fellowship, usmle step 1 pass/fail, virtual interviews

## Abstract

The application process for medical training fellowships has evolved significantly over the last decade. Important changes in access to quantitative data, the standardized application itself, and an unprecedented shift in the interview process have forced fellowship leaders to alter their evaluation process. This study aimed to assess each of those aspects of the pediatric cardiology fellowship application process. We review the application itself, focusing on the Experiences section of the Electronic Residency Application Service (ERAS) evaluation and the Pediatric Cardiology Fellowship Evaluation Form, which accompanies each letter of recommendation (LOR). In addition to these novel analyses, we present peer-reviewed literature on the topics of transitioning to pass/fail scoring for USMLE Step 1, declining provision of medical school class rank (especially among top-tier institutions), and the abrupt pivot from in-person interviews in favor of a virtual format. To accomplish these goals, we conducted a retrospective, mixed-methods analysis using three primary approaches. We performed a quantitative assessment of word counts in the Experiences section of ERAS applications submitted to a large pediatric cardiology fellowship program from 2014 to 2016 and compared them with those from 2024 to 2025, a decade later. We analyzed the Pediatric Cardiology Fellowship Evaluation Form for 138 applications, encompassing 2,680 individual ratings across six competency domains. Finally, we examined peer-reviewed research on the loss of objective performance measures in medical school and the Step 1 exam as well as literature on in-person vs. virtual interviews.

We found a 78% increase in the median number of words per experience entry in the ERAS application, creating an administrative burden for reviewers, especially in large programs. Evaluation Form data revealed dramatic score inflation, with the applicant pool having to be substantially superior to their peers to warrant the scores received. While the data are mixed, there is a large body of literature raising concerns that the removal of Step 1 numeric scores, declining reporting of medical school class rank, and the loss of in-person interview experiences have together compromised educational leaders’ ability to accurately assess applicants' qualifications for their training programs. These findings highlight a disconnect between both quantitative data and face-to-face interactions that could be beneficial for a fellowship selection committee and raise concerns about how changes in application and recruitment practices may affect both leadership and trainees in advanced medical training.

## Introduction

Selecting high-quality applicants for enrollment into advanced medical training is a difficult but critically important task. Owing to myriad factors, from a worldwide pandemic to shifting discussions about equity and balance, there have been substantial changes for pediatric cardiology fellowship applicants and program leadership. In this study, we review several notable changes to the application process for pediatric residents applying to a pediatric cardiology fellowship. We analyze the Experiences section of the standardized application and the Pediatric Cardiology Fellowship Evaluation Form that accompanies Letters of Recommendation (LOR). Using peer-reviewed literature, we examine the decline in objective data available to pediatric cardiology fellowship leadership as they seek to identify applicants who best fit the needs of their individual programs. We highlight the change to pass/fail scoring of the Step 1 USMLE, decreased reporting of class rank by medical schools, and the shift from in-person to virtual interviews for fellowship recruitment. Finally, we consider how these changes may impact fellow selection.

All pediatric residents are required to submit their applications through the Electronic Residency Application Service (ERAS), a national application service that allows for standardization of the application. To investigate the changing landscape of the ERAS application, we chose to focus on the Experiences section, which includes applicant experiences in research, work/employment, and volunteerism. This section comprises a robust portion of the application and gives the applicant the most latitude in presenting information to reviewers. Since the 2023 application cycle, ERAS has allowed applicants to label up to three experiences as those that were most meaningful to them. Once labeled as such, applicants are allowed to write a more detailed explanation of how the experience impacted them, and that explanation is included as a supplement to the standard entry.

Accompanying each LOR for pediatric cardiology fellowship is a Pediatric Cardiology Fellowship Evaluation Form, which allows the recommendation writer to select a box corresponding to the applicant’s relative level of proficiency across six domains. The domains are as follows: overall clinical ability, interpersonal skills, intellectual skills, potential as a clinical cardiologist, potential for research, and leadership. For each of these domains, authors may select one box corresponding to the following skill levels: superlative (upper 5%), outstanding (upper 10%), very good (upper 20%), average (upper 50%), below average (lower 50%), and unable to judge. The form is publicly available on the ERAS website [1]. We sought to answer the question of how good a cohort of applicants would have to be to warrant the evaluation scores we received.

Currently, there is renewed public interest in the student selection process. The University of California, San Diego (UCSD), a competitive undergraduate institution, recently reported that between 2020 and 2025, there was a 30-fold increase in freshmen who lacked high school-level math skills, and 70% of those were even below middle-school level [2]. Among other factors, administrators point to two topics covered in this manuscript - the elimination of standardized testing and grade inflation. They found that, among all available student data, the math SAT score is the best predictor of math placement at their institution. If eliminating standardized examinations contributed to a decline in the accurate assessment of college student aptitude, we should consider how the transition of USMLE Step 1 to a pass/fail format since 2022 has influenced recruitment in graduate medical education. An important distinction between the SAT and USMLE series of tests should be emphasized - high school students can take the SAT multiple times, reporting only their best score (or even their best combination of scores) to universities. Medical students applying to residency have only one scored exam, the USMLE Step 2, available at the time of residency applications. Because students are allowed to take the exam only once, there is tremendous pressure to perform in a single exam sitting. With the USMLE Step 1 score no longer available, educational leaders may look for other comparative metrics, such as class ranking. The impact of these adjustments is still being determined. We conclude with a review of some adverse effects surrounding perhaps the most dramatic change in the medical education application process - the abrupt shift to virtual interviews.

Our goals for this work are as follows: (1) to quantitatively assess temporal changes in the ERAS Experiences section verbosity, (2) to quantify score distribution of Pediatric Cardiology Fellowship Evaluation Forms, and (3) to contextualize these findings with a targeted narrative review of current literature pertaining to the decline in both objective application metrics and in-person interactions during recruitment.

## Materials and methods

Within this evolving environment, there remains a need for empirical data describing how these changes manifest in actual fellowship applications. The current study, therefore, focuses on two quantifiable elements of the pediatric cardiology fellowship application: (1) changes in the ERAS Experiences section, comparing the number of words per entry in applications submitted by fellows who matriculated between 2014-2016 with those from the 2024-2025 application cycles. (2) The distribution of scores on the ERAS Pediatric Cardiology Fellowship Evaluation Form during the 2025 cycle.

To investigate changes in the Experiences section of the ERAS application over time, we reviewed 14 standardized applications from pediatric residents who were accepted to our pediatric cardiology fellowship program approximately 10 years ago (2014-2016) and compared them to 14 applications from residents accepted to our program from the 2024 to 2025 application cycles. We included the application for all 14 accepted residents from 2024 to 2025. Four application records from the 2014 to 2016 cohort were incomplete and were excluded. From the remaining 16 complete applications from the 2014 to 2016 cohort, we randomly selected 14 applications for analysis. We counted the number of words in the Experiences section for every application by copying all text from the Experiences section and pasting it into Microsoft Word, then using the word count feature. We calculated the applicant-level mean words per entry and compared them using the Mann-Whitney U analysis. Group medians and interquartile ranges (IQR) were reported. To ensure that any increases in word count were not attributable to the explanations that accompany the new “most meaningful” addition to the application, we did a second Mann-Whitney U analysis with the “most meaningful” word count removed. Workload impact was determined by calculating the difference in the median word count between the two cohorts, the median number of experiences listed, and extrapolating that across the 141 applications (the number our program received in 2025).

We analyzed the Pediatric Cardiology Fellowship Evaluation Form scores and found the lowest-scoring form using quantitative methods. To determine how highly performing the group of applicants would need to be to justify the evaluations we received, we performed a chi-square goodness-of-fit test. We compared the scores applicants received to scores that would occur if a hypothetical pool of applicants comprised the top 25% of their residency class. The theoretical top 25% was prespecified, hypothesis-driven, and chosen a priori for analysis, given the competitive nature of pediatric cardiology fellowship applications, with the hypothesis that applicants to our subspecialty may perform better than average pediatrics residents. The hypothetical applicant distribution assumes that each rating is an independent observation and that letter writers use the explicitly stated percentile descriptors for scoring. The theoretical distribution did not incorporate observed data.

To contextualize other significant changes in the fellowship application process, we conducted a targeted narrative review of the peer‑reviewed literature in the following three domains that directly affect fellowship applicant evaluation: (1) the transition of USMLE Step 1 to pass/fail reporting, (2) declining availability of medical school class rank, and (3) the shift from in‑person to virtual interviews. We used PubMed keyword searches, citation chaining, and the similar articles function to identify studies that elucidate the challenges posed by policy changes. We prioritized recent literature while including older, foundational work when necessary. As stated in the limitations section, many articles report contradictory findings. Given that this writing is intended to be a targeted review, they are beyond the scope of this work and were, therefore, excluded. For some of the most current topics, online reporting from established organizations with editorial oversight was included.

## Results

**Figure 1:**
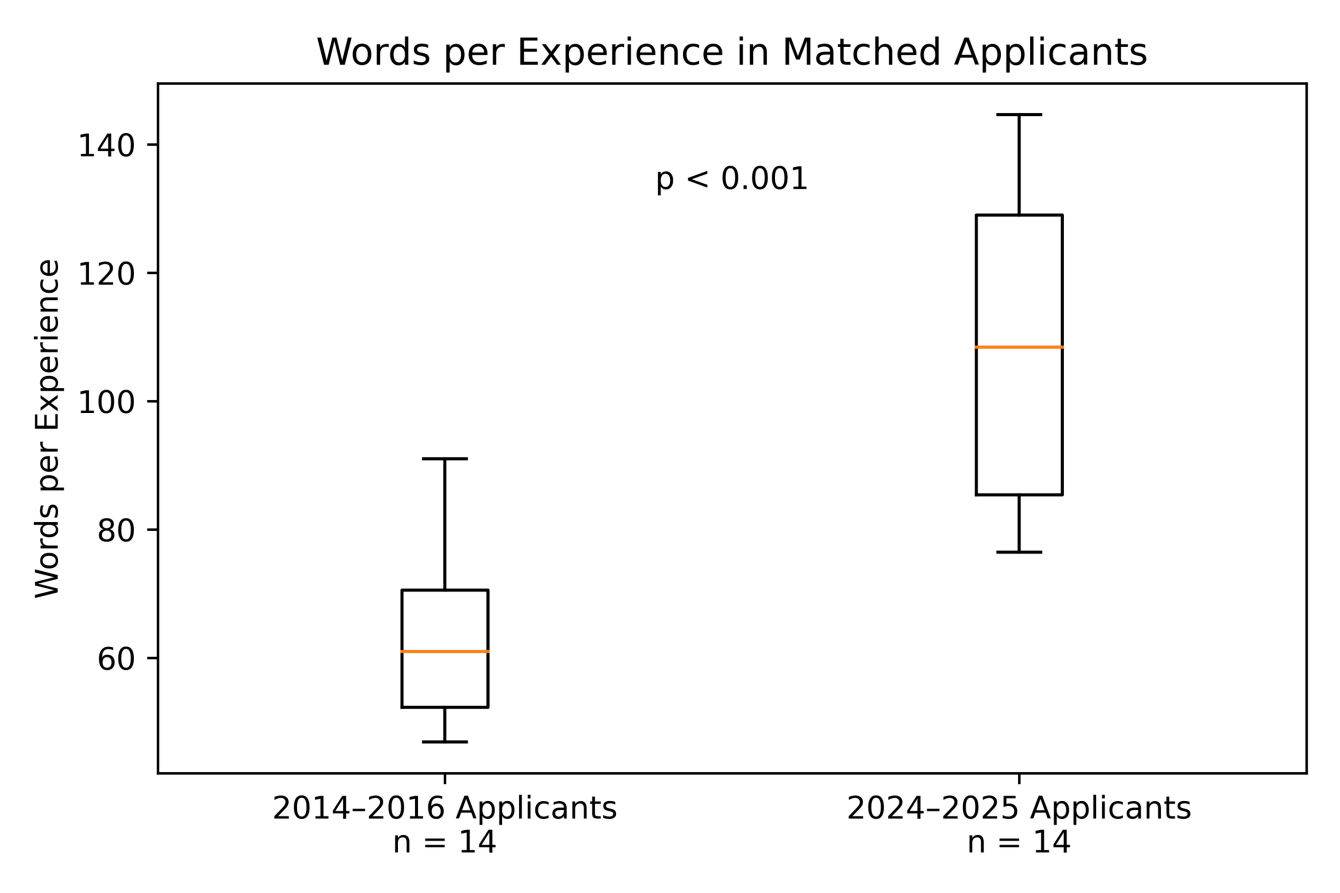
Box-and-whisker plot comparing median words per experience entry in ERAS applications from matched applicants in 2014-2016 vs. 2024-2025. ERAS: Electronic Residency Application Service

When comparing 14 standardized ERAS applications from residents accepted to our program 10 years ago (2014-2016) with 14 from 2024-25, we found that the use of words in the Experiences section has expanded significantly. The median number of words used by applicants to describe each listed experience in the modern cohort was 108 (IQR: 85.4-129) compared to 61 (IQR: 52.3-70.6) 10 years ago (p<0.001) (Figure 1). This is a 78% increase in the median number of words per experience. With a median of 10 experiences per application, extrapolated to 141 applications, the difference amounts to 66,834 more words, or about 134 additional pages of single-spaced type using 12-point font. When removing words added to explain the “most meaningful” experiences, there was a median of 93.1 (IQR: 68.7-115.9) words per experience used in 2024-25, a 53% increase from a decade ago (p=0.01).

**Figure 2:**
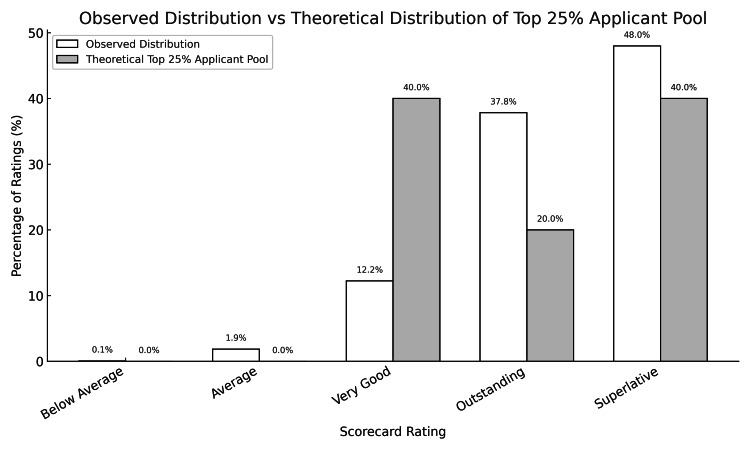
The distribution of actual Pediatric Cardiology Fellowship Evaluation Form scores received compared to the distribution that would be expected for a theoretical top 25% pediatric resident applicant pool.

Of the 141 ERAS applications submitted to our program in 2025, 138 contained at least one Pediatric Cardiology Fellowship Evaluation Form. We found that the actual scores given to the pool of pediatric residents applying to pediatric cardiology fellowship in 2025 were so skewed toward superlative that even if the applicants performed at the top 25th percentile of their residency classes as a group, the scores given would still not be statistically justified (p<0.001) (Figure 2). Further analysis of these data is available as a preprint [3]. The single lowest-scoring applicant to our program received the following scores across the following three scorecards: below average (1), average (6), very good (9), outstanding (2), superior (0).

We reviewed 14 articles related to the adoption of pass/fail scoring for the USMLE Step 1, 14 publications discussing the shift to providing medical school class rankings, and six publications on the transition from in-person to virtual interviews (Table 1). Some articles covered multiple topics.

**Table 1:** Key published findings on USMLE Step 1 pass/fail, MSPE/class rank, and virtual vs. in-person interviews in applicant selection. CK: clinical knowledge; IMGs: international medical graduates; PD(s): program director(s); USMLE: United States Medical Licensing Examination; ABOS: American Board of Orthopedic Surgery; GPA: grade point average; RR: relative risk; MSPE: medical student performance evaluation; CI: confidence interval

Studies	Topic	Key statement (as reported)
Ozair et al. [4]	Pass/fail	Exam-related anxiety is likely only to increase, as candidates now only have one chance to obtain a top score; this change has also removed the chance to demonstrate an improvement in scoring from Step 1 to Step 2 CK." "Importantly, given that IMGs have historically relied on high Step 1 scores for demonstrating their competitiveness in the residency match, the potential impact of this change cannot be overstated.
Asaad et al. [5]	Pass/fail	Eighty-three percent of respondents were strongly dissatisfied with the conversion to pass/fail reporting. Future emphasis will be placed primarily on subjective metrics, including applicant familiarity.
MacKinnon et al. [6]	Pass/fail	A majority of PDs (69.6%) disagreed that the change was a good idea, and a minority (21.6%) believed the change would improve medical student well-being.
Dougherty et al. [7]	Pass/fail	33% of those who scored 209 or less on Step 1 failed the ABOS Part I. USMLE Step 1 scores correlated with ABOS Part I certifying examination scores, and we therefore believe it may be used as one factor in resident selection.
Chen et al. [8]	Pass/fail, class rank	High USMLE scores, class rank in medical school, and interview performance were predictive of high examination scores in residency and good clinical performance. Class rank appeared to be the best predictor of scholarly publication and pursuing an academic career beyond residency.
Kenny et al. [9]	Pass/fail, class rank	The strongest positive associations referred to examination-based selection strategies, such as the USMLE Step 1, and examination-based outcomes, such as scores on in-training examinations. Moderate positive associations were present for medical school marks and both examination-based and subjective outcomes.
Filiberto et al. [10]	Pass/fail, class rank	Better performance as an intern was associated with higher USMLE scores, medical school GPA, and class rank.
Dutoit et al. [11]	Pass/fail, class rank	For applicants who do not provide a program signal, the results of this survey indicate that USMLE Step scores are one of the most important drivers of interview selection, followed by “other” factors. This is unsurprising as the amount of objective data by which to evaluate the large number of prospective applicants to residency training programs continues to decrease, particularly now that USMLE Step 1 results are “pass/fail” without a numerical score." "Medical school quartile or academic performance and geographic preferences were also selected as important by over 35% of programs.
Brancati et al. [12]	Class rank	Among academicians, higher attained rank in 1990 was independently associated with the following: (1) membership in Alpha Omega Alpha (RR=4.94, p=0.0001); (2) rank in the top third of the graduating class (RR=2.68, p=0.01); and (3) research experience in medical school (RR=3.11, p=0.0001).
Boysen et al. [13]	Class rank	There is extensive variation in ranking systems used in MSPEs. Program directors may find it difficult to use MSPEs to compare applicants, which may diminish the MSPE’s value in the residency application process and negatively affect high-achieving students.
Cottrell et al. [14]	Class rank	Removing objective data from the MSPE, however, may result in unintended disparities for students attending non-marquee schools or schools that have limited research opportunities. A holistic review does not mean that objective data should be purged. Rather, data like common quartile rankings complement other information shared in the MSPE, such as “noteworthy characteristics.”
Meyer et al. [15]	Virtual interview	The average number of programs each applicant applied to increased from 35.4 to 47.7 (p=0.002) when residency interviews switched from in-person to virtual. Negative themes included 38 (of 123) noted difficulty assessing program fit, 19 wanted to see the program or city in person, eight had increased interest in home/local programs, and six found it difficult to make connections or stand out.
Dalrymple et al. [16]	Virtual interview	Thus, applicants who interviewed virtually applied to nearly double the number of programs as those who interviewed in-person. The odds for a resident to receive an interview offer for a given application were 2.95 times (95% CI: 2.36-3.68, p<0.001) greater for the residents who interviewed in-person compared with the residents who interviewed virtually. Residents who interviewed in-person were more likely to express confidence in “getting a feel for the culture of a program” (76.9% very or extremely effective vs. 17.1% among virtual applicants, p<0.001) and in “getting a feel for the livability and quality of life of a program’s location” (56.0% very or extremely effective vs. 8.6% for virtual applicants, p<0.001).
O’Connor et al. [17]	Virtual interview	Nearly all who attended an in-person visit (96.5%, 109 of 113) found it valuable. All program directors liked the optional in-person visit and believe future applicants should be offered similar in-person visits. Most applicants who visited felt that they learned more about the program and the city from visiting, and 76.8% (86 of 113) reported that the in-person visit influenced their rank list.

## Discussion

Anecdotally, we noted over time that the Experiences section of the ERAS application has become longer and more time-consuming to review. We became concerned that the important message that the applicant was trying to convey about each experience was being lost in longer prose. Our analysis confirmed that there was a substantial increase in the number of words written per experience, with a 78% increase in the median number of words per experience entry. Though applicants may see these in-depth explanations as beneficial, educational leaders who are tasked with reading thousands of experience descriptions may come to the opposite conclusion. While our fellowship program has a relatively large number of applications for our specialty, 141 applications pale in comparison to the number regularly received by large pediatrics programs, which can easily exceed 1,000. The amount of time required to meaningfully review this part of the application has therefore increased tremendously, as supported by our workload impact results. The burden is compounded by the published finding that applicants are applying to more programs in the virtual interview era [15]. Equally concerning is that the message the applicants are trying to convey may be diluted by excessive detail. The lengthening of the Experiences section was not explained solely by the 2023 addition of the “most meaningful” experience. It remains to be seen whether this additional information has any effect on an applicant’s candidacy.

Analyzing the Pediatric Cardiology Fellowship Evaluation Form, we found evidence of tremendous score inflation. If the pool of cardiology fellowship applicants is actually average, compared to their peers in pediatric residency, the chance that the scores applicants receive accurately reflect their abilities is infinitesimally small. Even if applicants to pediatric cardiology as a group represent the top quartile of residents, there is still mathematical evidence of marked score inflation. Despite the recognition that applicants will request scores only from faculty who they think hold them in high regard, the dramatic skewness of the data cannot be ignored. We do feel that there is a role for these scores in the ERAS application and that educational leaders will benefit from the knowledge that such skewness exists. Further analysis of score data has been performed and is currently in press [3].

USMLE Step 1 scores were a fundamental part of residency and fellowship applications, with the large majority of program directors using them as a factor in recruitment [4]. In a study of anesthesia residencies, step scores were the most important driver of interview selection for applications when signaling of interest was absent [11]. There may be justification for using the metric in this way, as some data show correlation between USMLE Step 1 scores and both residency in-training exam scores and subspecialty board exam scores across a number of fields of medicine [4,7,18,19]. In particular, low Step 1 scores are correlated with board exam failure [20]. Since January of 2022, the USMLE only reported whether a test taker passed or failed Step 1, removing a marker of future performance, including risk for board failure. This is particularly important for program directors, where board passage is not only a critically important outcome for the physician’s career, but it is also a quality metric tracked by the American College of Graduate Medical Education (ACGME). The loss of this data point has been met with disapproval from program directors [5,6]. With this, many educators have made the obvious shift to considering the Step 2 Clinical Knowledge exam more heavily or on other application metrics, such as LOR strength and national ranking of the applicant’s medical school [5,21,22]. In a meta-analysis of 80 publications and over 41,000 participants, exam-based metrics were most strongly associated with in-training evaluation reports (ITER) [9]. They found that the Step 1 effect size on ITER was less than that of Step 2 and medical school reputation, but greater than that of the interview and reference letters. Thus, program leadership no longer has access to a measure more strongly correlated with training success than interviews and letters of recommendation, both of which are highly valued. Admittedly, the data are mixed on this topic. Today, USMLE Step 2 is the one and only quantitative standardized metric applied to every applicant. With only a single opportunity to demonstrate their fund of knowledge, the significance of one exam is substantially increased. Even medical students are not unified in their opinion of the Step 1 pass/fail policy. In 2020, prior to the change, 39% of students surveyed were in favor, while 31% opposed [23]. Opposition was most strongly felt by students interested in highly competitive residencies. A total of 73% reported that they would study less for Step 1, shifting that time to studying for Step 2 and conducting research. Interestingly, the passage rate for Step 1 has decreased among first-time MD test takers from 95% in 2021 to 91-93% in the pass/fail era [24]. This lower rate has been attributed to a higher passing score requirement and decreased pressure to study. As such, some students have suffered from the change. Further, a failing score on any USMLE exam used to be marked and readily evident to reviewers in the ERAS application under the exams tab. Now, application reviewers are required to download and open a separate PDF file in the documents tab to find for themselves whether the applicant failed any of the exams. This makes a very important data point less accessible and comes at the cost of extra clerical work for reviewers. Given the findings above, it is logical for the community of graduate medical school educators to consider the utility of quantitative scoring in this context. Of note, other institutions of higher learning, like the Massachusetts Institute of Technology (MIT) and almost all Ivy League colleges, have resumed the requirement for standardized testing [25]. Even UCSD, currently part of the test-blind University of California system, has recommended consideration of the same [2].

Medical school performance is a common consideration in residency and fellowship selection [11]. In the previously cited large meta-analysis, the authors concluded that, “medical school grades and standardized test scores are among the applicant items with the strongest positive associations currently available for resident selection committees” [9]. High medical school class rank was the variable most strongly associated with anesthesiology fellowship performance, scholarly publication, and pursuit of an academic career beyond residency [8]. Early and late physician performance outcomes have been found to be related to medical school GPA, as studies demonstrated a positive correlation in intern year as well as after two decades of practice [10,12]. Despite this evidence, there has been a gradual decrease in reporting of class rankings by medical schools, which instead provide a summative written assessment in the form of a Medical School Performance Evaluation (MSPE). In a sample from 2012 to 2015, three-quarters of schools provided student rankings in the MSPE, while about two-thirds did so only five years later [13,26]. While almost all medical schools report graduating students’ clerkship grades (98.6%), far fewer report preclinical grades beyond pass/fail (30.8%) [26]. This is particularly true for schools considered “top-tier.” In a 2023 study, no school ranked in the top 10 for reported preclinical grades, whereas 17% of schools ranked 11-50, 52% ranked 51-100, and 59% were unranked [27]. Further, both MSPEs and grades are highly variable across schools, making comparison difficult or impossible. Even key words like “good,” which have traditionally carried meaning related to class rank (bottom 25% in the case of “good”), are inconsistently applied. Like preclinical grades, summative assessments that compared graduating students to their colleagues were absent in schools ranked in the top 10 [27]. As school ranking increased, so did the likelihood that class ranking was reported, raising questions of fairness. A low-achieving student at a highly-ranked school has a tremendous advantage, as their actual performance is shrouded. All this has led to a call for accurate and fair student comparisons in the MSPE, with a concerted effort to eliminate variability and ambiguity [14]. As stated by Cottrell et al., “holistic review does not mean that objective data should be purged" [14]. Reporting medical school ranking may provide some benefit to educational leaders who consider applications in their entirety.

The shift from in-person to virtual interviews may be the most significant change in graduate medical education in the last decade. While there has been nearly ubiquitous adoption of this practice, the current climate seems to be shifting back to offering at least the option of in-person experiences for applicants. Satisfaction with virtual interviews was higher among applicants (69%) compared to interviewers (44%) for family medicine residency [28]. There was a strong preference among both groups (72% of interviewees and 81% of interviewers) for some form of in-person option, whether required or voluntary. While one large study recently reported high (and increasing) support for continuing virtual interviews among residency applicants, it did not also ask about interest in an in-person option [29]. When studying that option specifically, another article reported that almost all internal medicine residency applicants (97%) who visited a program in person found the experience to be valuable in informing their decision, and 100% of PDs liked having in-person visits [17]. In some ways, it may be in the applicant’s best interest to interview in person. Those who interviewed for neurology residency in person were more confident that they understood programs’ cultures compared to those who interviewed virtually (77% vs. 17%), and despite the time and financial requirements of doing so, 96% of in-person interviewees preferred that modality [16]. While this may prove to be a beneficial change for applicants, the implications for residency programs must also be considered. Residency programs are tasked with matching individuals who will best fulfill the roles and responsibilities of their training environments, and their needs and values should therefore be weighed just as strongly. If, as one study demonstrated, resident fit with a program, commitment to specialty, and interpersonal skills cannot be fully assessed by program directors during virtual interviews, educators are placed at a distinct disadvantage [30]. Requiring programs to finalize their rank lists before allowing applicants on campus removes any evaluatory benefit the programs stand to gain, even as some programs choose to fund part or all of those visits. There is still more potential advantage in leaving the rank lists open for adjustment. Some PDs found that applicants had better communication skills in person than they anticipated based on their virtual interviews [17]. As medical training is a decidedly in-person activity, consideration should be given to allowing applicants the opportunity to interview in person.

There are several limitations in our study that should be acknowledged. Given a single-institution design, restricted access to ERAS data, and incomplete historical data, sample sizes are small. This limits reproducibility, representativeness, and external validity. Because we only had historical data on accepted residents (not all resident applicants), the true variability of applicants may be underestimated. Data on residencies provide a more robust sample; however, we felt that it was important to represent our specific cohort in this study. Different institutions will review the ERAS application in unique ways, making the word count more impactful for some and less for others. The top 25th-percentile benchmark is a hypothetical distribution that has not been empirically validated. While we present peer-reviewed data, there are conflicting studies on every topic in this space, including pass/fail USMLE scoring, provision of medical school class ranking, and in-person interviews. We acknowledge the variability in data and the diverse opinions of both the educators and learners involved in this process. We recognize that many are divided on these topics, citing important considerations like financial concerns, diversity, equity, and inclusion. While not a systematic review, our narrative work endeavors to respect those considerations while highlighting data from a diverse cohort across many training levels and disciplines. Recognizing these limitations, inferences from these data and their impact on policy considerations must be considered judiciously.

## Conclusions

Our novel data quantifies the increase in verbosity in the Experiences section of the ERAS application, as well as inflation in evaluation forms. Our focused literature review highlights a decline in the availability of quantitative data for training program leadership and selection committees. Combining these findings with the inability to meet applicants in person prior to finalizing a rank list, leadership faces some challenges in the current process. This has led to some dissatisfaction among program directors. We believe that using word limits in the Experiences section of the ERAS application may benefit both applicants and reviewers. By increasing awareness of score inflation, perhaps letter writers will be more objective in the future, and this modality may achieve its intended potential. We see a role for quantitative metrics in the application process. Allowing in-person interviews or visits after which directors are allowed to adjust their rank lists based on these very important interactions could be considered and may benefit applicants and directors alike. After all, it is these types of interactions that will occur in every training experience that will follow. With ongoing refinement, we are optimistic that the graduate medical education application process will continue to improve.
